# Formulary for the Preparation and Employment of the New Medcines Veratria, Hydrocyanic Acid, Delphinia, Iodine, &c.

**Published:** 1828-05

**Authors:** 


### REVIEWS AND BIBLIOGRAPHICAL NOTICES.

---

ART. II.—*Formulary for the preparation and employment of many new Medicines; such as the Nux Vomica, the Salts of Morphine, the Prussic Acid, the Strychnine, the Veratrine, the Alkalis of the Cinchona, the Emetine, the Iodine of Mercury, the Cyadine of Pottassium, the Croton Oil, the Salts of Gold, the Salts of Platina, &c. &c. By* F. MAJENDIE, *Member of the French Institute, Titular of the Royal Academy of Medicine and of the Philomatic Society, Physician of the Central Office of Admission to the Hospital and Almshouses of Paris, &c. &c. Translated from the fifth edition, revised, and augmented, by* JOHN BAXTER, M. D. *Member of the Medical Societies of Philadelphia and New-York. With notes and abbreviations. New-York,* 1827. *pp.* 138.

[Concluded.]

VERATRIA. The next, of the new principles on which our author treats, takes its name from the *Veratrum Commune*, or White Hellabore, and the *V. Sabadilla* or Cevadilla, in both of which it exists. It is found, also, in the root of the *Colchicum Autumnale.* Like the others it possesses alkaline properties, and dissolves in all the vegetable acids, with which it forms neutral salts, that are uncrystallizable. Veratria is nearly insoluble in water, but dissolves readily in alcohol and ether. Its operation and effects on the living body may be collected from the following extract:

"*Action of veratrine upon animals.* A very small quantity of acetate of veratrine, injected into the nostrils of a dog provokes immediately a violent sneezing, which lasts sometimes near a half an hour.

One or two grains thrown in the throat determine immediately a very abundant salivation, which lasts some time.

If the same quantity of this substance be injected into any point of the intestinal canal, and the abdomen opened to observe its effects, the intestine will be seen to be much hardened, then to relax, then to contract again, and thus to continue for a certain time. The part of the mucous membrane which is found in contact with the veratrine is inflamed; the irritation spreads and determines vomitings and alvine evacuations. Given in a larger dose, it produces a great acceleration of the circulation and of respiration, soon followed by tetanus and death.

The effects are still more rapid, if we inject under the pleura or into the tunica vaginalis one or two grains of this substance: in less than ten minutes we see death take place after the tetanic phenomena.

The same quantity injected into the jugular vein produces, also, but in a few seconds, tetanus and death. Autopsy of the body shows that, even in this case, the veratrine has exerted an action upon the intestinal canal, the mucous membrane of which is found highly injected. The lungs, also, show signs of inflammation and engorgement. After what has been given above, we see, that this substance, thrown in small quantities into the intestinal canal, produces only local effects, or at least effects confined to the intestinal canal, and that it is necessary that it should be administered in a large dose, or thrown upon parts where absorption is very active, such as the pleura and tunica vaginalis, to produce general effects, which we have just shown are so terrible. M. Andral (son) has published a note on the action of veratrine, in the first number of my Journal de Physiologie.

*Action of veratrine on the well and on the sick.* The effects of veratrine in large doses have not been observed upon man: without doubt they would be the same as those observed upon animals.

The taste of veratrine is very acrid, but without any mixture of bitterness; it excites, however small the quantity may be that is taken into the mouth, a very abundant salivation.

Although the veratrine is absolutely inodorous, there is an inconvenience, when it is in a pulvulurent state, from smelling it too near. The small quantity carried into the nostrils by the air is sufficient often to promote violent sneezing, which may become dangerous.

Thrown in the dose of a quarter of a grain into the intestinal canal, it determines promptly very abundant alvine evacuations: in a little larger dose, it provokes vomitings more or less violent.

I have given it lately in the dose of 2 grains in twenty-four hours, without obtaining too abundant evacuations. The patient was an old man who had been taken with appoplexy some time before. It is a new proof that the state of the nervous system influences medicines very much in the manner of acting.

After having tasted with care the potion which contained these two grains of veratrine, I experienced for several hours an insupportable acridness in the mouth and pharynx. The impression had not entirely gone the next day. The patient experienced nothing of the kind.

*Cases in which the veratrine may be employed.* This substance, producing the same effects as the plants from which it is drawn, may be substituted for them, and with much advantage, since we know in this case what we are ignorant of in the other, the quantity of active substance which we make use of.

The veratrine answers, moreover, to excite promptly, in cases where it is necessary, full alvine evacutions: given with this intention, it has succeeded very well in certain old men, in whom there existed an enormous accumulation of fœcal matter in the large intestine. In the pharmaceutic preparations of which the colchicum and the hellebore form the base, the veratrine may replace those substances; they will become then therapeutic agents more powerful, more convenient, and more sure. The pills of Bacher, l'eau medicinale d'Husson, the simple tincture of colchicum, will cease to be remedies unbelieved in and too often complained of by practitioners.

The following are some formulas which we have written out, to replace those of which we have just spoken:

*Pills of veratrine.* ℞ Veratrine, 1-2 grain,
Gum arabic and syrup of gum, a sufficient quantity to make 6 pills of 1 grain each. One of these pills may first be given, and if purgative effects are not obtained, 3 may be given in a day; they will replace with advantage the pills of Bacher.

*Tincture of veratrine.* ℞ Veratrine, 4 grains,
Alcohol, 1 ounce.
This tincture is administered in the dose of 10, 15, 20, and 25 drops in a cup of good drink. It may be given internally with advantage, instead of the tincture of colchicum, in dropsy, in leucophlegmasia and anasarca; and, to the exterior, by friction, in those same diseases and in gout.

*Solution of veratrine.* ℞ Sulphate of veratrine, 1 grain,
Distilled water, 1 ounce.
This solution may replace the l'eau medicinale d'Husson.

*Ointment of veratrine.* ℞ Veratrine, 4 grains,
Axunge, 1 ounce.
This ointment may be employed externally in cases of chronic rheumatism, anasarca, and in gout." pp. 57—58—59—60.

We have not prescribed Veratria or its salts, except as it exists in the vinous tincture of Colchicum. That preparation we have repeatedly employed in bronchial inflammation, and can testify to its efficacy. After bloodletting, it speedily diminishes the frequency and force of the pulse, with an abatement of dyspnœa. It may be given alone, in a concentrated state; but is more efficacious, when added to a dilute solution of tartarized antimony, with laudanum.

Hydrocyanic or Prussic Acid. Of the whole Materia Medica, this is one of the most deleterious and decomposable substances. To these two characteristics we may ascribe its limited employment by the profession at large; to whom it has been so strongly recommended by several distinguished physicians of France, England, and the United States. Its tendency to decomposition is so great, as to preclude all certainty as to the quantity actually administered; and a knowledge of this, has often deterred both physicians and patients from its use. Those only who live in the vicinity of the laboratory can, indeed, employ it with safety and success. When diluted with distilled water, we have found the difference in strength so great, between a recent solution and one a few days old, as to constitute a serious embarrassment in practice. One of our patients, in fact, came nigh being injured, by taking no more of a solution, just prepared, than she had been accustomed to take of one that had been kept a week or two. It produced instantaneous reduction of muscular energy, to such a degree, that she nearly fell upon the floor. Notwithstanding this most striking effect of the Prussic Acid, we have found it, in moderate portions, to increase the excitement of the nervous system, and raise the spirits of dyspeptic patients.

Of the potent agents which the laboratory has sent forth, in these latter days, not one has given rise to so many volumes of reports, as that under consideration. We do not believe, however, that it has ever cured either Dyspepsia or genuine Phthisis Pulmonalis, the diseases for which it has been most lauded. This is the result of our trials with it, in no inconsiderable number of cases—the medicine being recently and accurately prepared, by the ingenious Dr. Best, late lecturer on Pharmacy in Transylvania University. In both maladies, however, and in many imitative phthisical disorders, we have found it a valuable palliative. Few substances exert so much influence over that morbid sensibility, especially of the animal system, which constitutes so prominent a circumstance in Dyspepsia, and many sympathetic affections of the lungs and muscles of respiration. Some of these cases assume a threatening aspect; and are greatly relieved or even cured, by the Prussic acid. Beyond this, its powers have not appeared to us to extend.

From its efficacy in subduing unhealthy sensibility and irregular action in the muscular system of animal life, we have been induced, in obstetrical practice, to employ it for *After-pains*, but could not discover that it exerted an influence on the contractions of the uterus. Its effects on the muscles of organic life, seem, indeed, to be inconsiderable.

The following quotation contains a summary of what our author has been able to collect, relative to the medicinal employment of this powerful agent:

"*Cases in which it should be employed.* The prussic acid, weakened, as we are going to show, is employed with success in all cases where the irritability of the pulmonary organs is morbidly increased: thus it is used advantageously in the treatment of nervous and chronic coughs, in asthma, whooping cough, in the palliative treatment of phthisis. A great number of observations now induce the belief that it can procure a complete cure when the disease is yet in its first degree. In England, it is employed with success against the hectic cough, sympathetic of the affections of another organ, and against dyspepsia. Mr. Elliotson, at the hospital of St. Thomas, in London, and in his particular practice, has employed the medicinal prussic acid; it was prepared according to the process of M. Vanquelin, which we have given above; he has reported more than forty cases of dyspepsia, with or without vomitings, and accompanied with sharp pains in the epigastric region, and of pyrosis, which were cured by the use of the medicinal prussic acid. This same physician cites a case of painters colic, in which Dr. Prout gave the prussic acid and procured instantaneous relief. Mr. Elliotson has also administered the hydrocyanic acid in a great number of affections of the chest; he has almost constantly obtained a cessation of the cough which fatigued the patient. Externally, the medicinal prussic acid, employed in lotions for different diseases of the skin, has not produced in the hands of Mr. Elliotson very marked effects; however, Dr. Thompson assures, that he has used it in lotions, with constant success, to diminish those itchings and burnings so fatiguing in cutaneous diseases, and to have cured several kinds of dartre, especially several persons affected with pimples on the face. (Acne rosacea.) M. Jacob Bouchenel has published an interesting memoir, on the use of the prussic acid in the treatment of chronic pulmonary catarrh, a memoir in which this physician reports four cases of chronic pulmonary catarrh cured by the hydrocyanic acid. The author closes by saying, that this remedy, employed in small dose, has no more inconvenience than a common draught; that the acute state of catarrh is not proper to use this medicine in, and that the success is more certain when antiphlogistic means are resorted to before employing the hydrocyanic acid. M. Bouchenel has also employed the prussic acid in a case of phthisis; but he attained only to calm momentarily the cough, which leads him to doubt whether the prussic acid has really procured the cure of phthisis. We repeat here, that we have obtained the cure of persons having all the signs of phthisis in the first degree, and even more advanced. It is not a chance assertion on our part.

In Italy, the medicinal hydrocyanic acid is used to calm the too great irritability of the uterus, even in cases of cancer, and to moderate the activity of the heart in almost all sthenic diseases.

Professor Brera speaks very much of the happy effects of prussic acid in pneumonia; he advises it also against rheumatism, and as a vermifuge. It is since the professor has employed the hydrocyanic acid in diseases of the heart, that Dr. Maclead administered it in these same diseases; (Bullet. des Sciences Med., Feb., 1824.) He states that he has calmed by this acid nervous palpitations, those especially which seem to have a derangement of the digestive organs for the cause; he has also used it as a palliative in some cases of aneurism of the heart. He has never carried the dose beyond 28 drops in twenty-four hours, and he has seen in no case serious accidents result from the administration of this remedy.

Dr. Frisch, physician at Nyborg, in Denmark, has calmed intolerable pains, caused by cancer in the breast, and which had resisted all antispasmodics, by washing the surface of the cancerous ulcer with dilute medicinal prussic acid. He has also employed this remedy usefully in several cases of phthisis. He cites a case of cure. Dr. Guerin, of Mamers, has lately employed with success the medicinal prussic acid in two cases of cerebral fever." 64—65—66.

Cyanide of Potassium and Hydrocyanite of Potash.—The perishable constitution of the Hydrocyanic or Prussic Acid, has suggested to the chemists, the value of some other and more permanent compound, of which Cyanogen should be the active ingredient. This has been discovered or invented, by Messrs. Robiquet and Villerme; and consists in the union of Cyanogen with Potassium, constituting a Cyanide of Potassium, which kept dry undergoes no change. Exposed to moisture, or dissolved in water, the Hydrocyanic Acid and Potash are formed, and there results a solution of Hydrocyanite of potash. The effects of these substances, as far as they have been ascertained, are stated by one author in the following paragraphs:

*Action of the cyanide of potassium and of the hydrocyanate of potash upon other animals and upon man.* Messrs. Robiquet and Villerme have made experiments upon animals in our presence.

The action of the cyanide of potassium was such, that with the tenth of a grain of that salt a linnet perished in one minute; a little less than a grain killed a guinea pig in two or three minutes.

With the hydrocyanate of potash, a small drop not containing more than an hundredth part of a grain of cyanide in solution, made a cock linnet fall dead in about half a minute. A half gros, containing 5 grains of cyanide, killed a large sized dog in 15 minutes.—The symptoms of poisoning were similar to those produced by the hydrocyanic acid. There has been no occasion to study on man the symptoms caused by this substance.

*Manner of employing it.* The cyanide of potassium is dissolved in eight times its weight of distilled water, it is transformed into hydocyanate of potash. The cyanide mixed with water in this proportion may receive the name of the medicinal hydrocyanate of potash.

We may then, without danger, give this hydrocyanate in the same doses as the medicinal prussic acid, and introduce it into the same preparations as those which are pointed out for prussic acid.—We may, besides, render it entirely independent of the action of the small portion of alkali contained in the cyanide, by adding a few drops of some vegetable acid, or by prescribing it with an acid syrup; from this will result the eminent advantage of putting the prussic acid more uncombined.

If the cyanide of potassium be put into a draught instead of the dydrocyanate of potash, we must commence with a quarter of a grain, and increase gradually to a grain, dose which has already been surpassed by some physicians." pp. 69—70.

Cyanides of Zinc and Iodine. Two other compounds of Cyanogen have been offered to the profession, as substitutes for the Prussic Acid. These are the Cyanides of Zinc and Iodine. Neither of them appears to be as energetic in its action on man, as the preparations of which we have spoken; but their effects remain to be ascertained.

Solania. This is the deleterious principle of the genus of Solanum, or Night-Shade. It was discovered by Defosses, a French apothecary, in the leaves and berries of the S. Nigrum (deadly night-shade) and S. Dulcamara (bitter sweet). The berries of the former contain it in abundance, in union with malic acid. The other species of Solanum have not been examined; but it probably exists in all the *active* members of the family. The following are its therapeutick properties, as far as they have been ascertained.

"*Action of solanine upon animals.* This substance, introduced in the dose of 2 to 4 grains into the stomach of a dog or cat, excites violent vomiting, soon followed by drowsiness, which lasts several hours.

A young cat was able to support without dying 8 grains of this substance. After violent vomiting, he experienced strong sleepiness, which lasted thirty-six hours.

*Action of solanine upon man.* If a small quantity of solanine is swallowed, a strong feeling of irritation is perceived in the throat.—Held in the mouth, the solanine gives a nauseous taste, slightly bitter, but which becomes very much so if the substance is dissolved in a little acetic acid.

Of all the salts of solanine, the acetate is the only one whose action has been tried on man. In the dose of a quarter of a grain, it produces nausea, but the tendency to sleep is not observable.

After what we have just said, we see that the solanine, like opium, may produce vomiting and sleep; but its emetic properties appear more developed than that of opium, while its narcotic properties are evidently much less.

*Cases in which they may be employed.* The solanine has not yet been tried upon the sick, but it may be used in cases where the extract of night shade or that of bitter sweet are indicated." p. 79.

Delphinia. A tincture of the seeds of the garden larkspur Delphinium Consolida et staphisagria, has long been in use among the people, to destroy lice, (Pediculi) an evidence that they possess deleterious powers. In 1819 M. M. Feneuille and Lassaigne discovered in them an alkaline element, on which their effects appear to depend. It has not yet, however, been made the subject of therapeutick investigation. In the United States there are several native species of Delphinium, which merit the attention of our pharmacopolists.

Gentiania. Two chemists, M. Henry and M. Caventou, at the same time, but distinct from each other, succeeded in extracting from the roots of the common Gentian (G. lutea) an active, bitter principle. Its alkaline properties are but feeble. Its best solvent is ether. It is yellow, inodorous, and has the aromatic bitterness of the gentian in a decided degree.

"*Action of gentianine upon man and other animals.* Some trials which I made, taught me that gentianine has no poisonous qualities:

Several grains of this substance injected into the veins, pro duced no apparent effect. I myself swallowed two grains dissolved in alcohol, and only experienced an extreme bitterness and a slight feeling of warmth at the stomach.

*Mode of employing gentianine.* The tincture is the preparation which should be most frequently used. It may be prepared from the following formula:

*Tincture of gentianine.* ℞ Alcohol at 24°, 1 ounce,
Gentianine, 5 grains.

This tincture replaces with success the elixir of gentian, and is employed in the same circumstances.

*Syrup of gentianine.* ℞. Syrup of sugar, 1 pound,
Gentianine, 16 grains.

It is one of the best bitters which can be used in scrophulous affections." p. 84.

Iodine. This simple substance, discovered by Curtois in 1813, has attracted an uncommon degree of attention. It was first detected in the sea weed which is used in the manufacture of soda; but has been since found in a great variety of marine plants and animals—such as shell fish and sponges; it exists likewise in sea-water, and in several mineral waters. It is remarkable, therefore, that it should not have been sooner discovered. Of mineral springs, the salino-sulphurous most constantly afford it. In the Western States these waters are abundant, and probably all contain this substance. None of them have yet been analysed with care; and they offer, therefore, an interesting subject of inquiry to such of our physicians, as are qualified for chemical researches. We should be much gratified to make public the results of their analyses.

Iodine, like Cyanogen, unites with Hydrogen and forms an acid, the Hydriodic, with which several neutral salts are formed. It also combines with Oxygen, and constitutes the Iodic acid. In sea and mineral waters, iodine exists in the state of a hydriodate of potash, and perhaps also of soda.

The interest which Iodine has excited among the physicians of the United States, will justify us in the following extended extract from M. Majendie's work:

"*Action of iodine upon man and other animals.* A little while after the publication of his handsome work on iodine, M. Gay Lussac sent me a certain quantity, in order that I might study its effects upon animals. I made immediately some experiments, in which I introduced the tincture of iodine into the veins, in the dose of 1 gros, without any apparent effect.

I made also several dogs swallow it, who vomited, but experienced no other effect.

Seeing this innocuity of the new substance, I swallowed myself a tea spoonful of this tincture, and there resulted nothing but a disagreeable taste, which remained several hours, but finally dissipated by little and little.

I have seen lately an infant four years old, to whom, by mistake, was given a tea spoonful of the tincture prepared by M. Pelletier; its lips and tongue were colored yellow, but no accident followed this event.

Besides the therapeutic properties of iodine, one of its most remarkable effects, when the employment has been continued for some time, is the dimunition of volume of the mammary glands in woman and of the testicles in man.

*Cases in which the preparations of iodine are employed.* M. Coindet, physician at Geneva, is the first who employed the iodine as a remedy; he used it in the treatment of goitre with very marked success. These attempts have been repeated since, as well in France as in Swisserland, by several physicians; and it seems to result, from their observations, that we have now in iodine an efficacious remedy against a disease which has sometimes shown itself so obstinate.

Although we ought especially to expect success from the employment of iodine when the goitre is recent, and in individuals who have not yet reached an advanced age, however, we have seen it dissipate old goitres, hard and voluminous; but, as in this case the treatment is necessarily longer, there may result from the long continued use of iodine an action injurious to the stomach: to remedy this inconvenience it has been sought to introduce iodine by another way, by that of frictions.

If new examples were necessary to prove to practitioners at this time the advantage of simple remedies over ancient formulas, we could cite the cases collected by Mr. William Rickwood, of goitres cured by iodine, while we have before used burnt sponge, but with only a partial success. (Lond. Med. and Phys. Journal, Aug., 1823.) Among the facts reported by this physician there is a curious one; it is the cure of a goitre, or at least a very considerable diminution of one, in a female aged 70 years.

Iodine has been employed in the treatment of scrophula with an equal appearance of success: a great number of cases have just now confirmed the utility of this remedy in that disease. M. Baup has cured, bv the use of iodine, old scrophulous ulcers. I have myself obtained, by this means, the resolution of very considerable glandular enlargements.

In the report on the Polyclinic institute of Berlin, for the years 1820, 21, and 22, Messrs. Hufeland and Osann, after having reported several cases of goitre cured by the tincture of iodine, and the hydriodate of potash, announce that some advantage has also been drawn from the same preparations of iodine, over scirrhus and carcinoma of the uterus. Dr. Wagner asserts that he has obtained good effects from iodine in the treatment of a tumor which he regarded as cancerous, and which was situated near the jaw. Doctor Hennemann (Journal der pratisehem heis Kunde) has also reported a case in which it seemed in fact that iodine had had a remarkable influence on a cancer of the womb arrived at the last stage; there was a communication between the vagina and the abdominal cavity, so that a cure could not take place; but the cancerous affection, says Dr. Hennemann, was much ameliorated.

M. Zinck, in 1823, read to the National Society of Lausanne, a memoir in which he reports two cases of white swelling cured by the preparations of iodine.

In the memoir of M. Gardiner on iodine, there is found a similar case of cure, which was communicated to him by Prof. Maunoir, of Geneva. A child had a considerable white swelling of the knee.—There was incapacity to walk without crutches: blisters, leeches, and resolvents of all kinds had been employed; friction was made morning and night on the tumour with a portion of iodine ointment as large as a nut; the tincture of iodine in the dose of 1-2 a grain at most was given internally. After some time the cure was complete.

M. Zinck has again published two memoirs (Journal Complimentaire, April and May, 1824) upon the abuse of iodine used internally, in which he has shown that the action of iodine prolonged for too long a time may bring on inflammation of the stomach; but this accident has only taken place from the abuse which the patients made of it from the hope to obtain a more prompt cure, or even to prevent the evil.

Lately the use of iodine has spread in England, where it has been used but by a very small number of Physicians. Dr. Gairdner has published an interesting memoir on the effects of iodine upon the animal economy, and on the advantages to be derived from it in the treatment of goitre, scrophula, and tuberculous affections of the lungs and abdomen. We would observe that it is very rare that iodine causes the serious accidents that are attributed to it by Dr. Gairdner: it is necessary that it should be administered with as little rule as it was by the young students of whom the author speaks, who wished himself to cure his sister of a goitre which she had. Dr. Baron, of London, appears to have administered iodine with some success in the treatment of scrophulous phthisis, and some other tuberculous affections. These first attempts require that we should collect new facts, that we may know to what point we may calculate upon the efficacy of iodine, when phthisis is but little advanced.

The late Mr. Haden, in his English translation of our Formulary, has also reported a case of phthisis, presumed cured by iodine.

M. de Fermon obtained very good effects upon a young woman with phthisis, by the following potion, which was taken a tea spoonful at a time every hour:

| | | |
|---|---|---|
| R. | Lettuce water, | 4 ounces, |
| | Solution of hydriodate of potash, | 15 drops, |
| | Medicinal prussic acid, | 10 to 15 drops, |
| | Syrup of marshmallows, | 1 ounce, |

Or the prussic acid and the syrup of marshmallows may be replaced by cyanic syrup.

This same Dr. Baron, whom we have just cited, reports in his Treatise on Tuberculous Diseases, a case of encysted dropsy of the ovary, in which the use of iodine was followed by the most prompt and most marked success. Dr. Gairdner, who cites this fact in his memoir upon iodine, and says that he had also advised it with great success in a similar case, prescribed it in several cases of acites, but obtained no good effect.

M. Coindet praises iodine as a powerful emmenagogue: this last property has been confirmed by the observations of Prof. Brera (Saggio Clinico sull' Iodio, Padoue, 1822) and of some other physicians.

M. Brera has even administered the preparations of iodine in the treatment of a much larger number of patients than M. Coindet has. To the cases of goitre and suppression of the menses cured by iodine, he has joined several cases of glandular indurations, tabes mesenterica, chronic dysentery, hemoptysis succeeding suppression of the menses, laryngeal phthisis, fluor albus, syphilitic enlargements, the cure of which he attributes to this same remedy. It may be that M. Brera too often joined other substances with the preparations of iodine, to which last he attributes the efficacy. We must not, therefore, employ these but with caution in similar cases. Further, M. Brera is not the only one who has given iodine for tabes mesenterica. M. Callaway, a distinguished English surgeon, has derived from the use of tincture of iodine the most happy results in scrophula and in enlargements of the mesenteric glands.

Iodine has been used lately for the treatment of syphilitic bubos and of blennorrhagia.

M. Richond, (Archiv. Gen. de Med., March, 1824, p. 322,) military physician of the hospital of Strasbourg, employed it with advantage in the treatment of these two diseases.

This physician gives generally in the morning 15 drops of the tincture, 20 to 25 the second, and 30 the third day. He commences, then, to give 15 drops at night, and augments in that way up to 30 drops morning and night. This dose is continued three or four days: finally, he carries the dose to 50 and 55 drops morning and night. If there be no irritation of the stomach, the patients experience sometimes a sentiment of burning in the pharynx; but this sensation soon dissipates: there are at times slight colics, headache, dryness and redness of the tongue; then suspend it for a short time. The dose most proper is 30 drops morning and night. M. Richond has only treated soldiers thus, the most part robust and little excitable.

Messrs. Gimelle (Revue Medicale, v. 7, p. 249) and Sablairolle have reported cases which will confirm the properties which Messrs. Coindet and Brera attribute to iodine against leucorrhœa. M. Gimelle has even cured dartres, by means of preparations of iodine. M. Eusebe de Salle has treated with success, by frictions of ointment of hydriodate of potash and iodine in pills, chronic enlargements of the testicles and chronic enlargement of the liver, which the English call "liver complaint," and which arise from the residence of Europeans in equatorial countries.

A veterinary physician, M. Roupp, attached to the depot of Stallions at Abbeville, wished to make use of the hydriodate of potash in the treatment of the glanders: he gave from 9 to 14 grains of the hydriodate of potash, for a month, to a horse, and had him rubbed with an ointment made with this salt This first attempt was without effect; it seemed even to augment the fever: perhaps the dose was too powerful. In the animal there was a too considerable disorganization of the lungs, of the pituitary membrane and of the bones which it covered, to hope for a cure. (Jour. Gen. de Med., April, 1824.)

At the end of the year 1822, the Geneva and Swiss physicians were much out of conceit of the advantages which they had at first thought to have reaped from the preparations of iodine: they pretended that serious accidents had followed their use, such as chronic inflammation of the stomach, and considerable and rapid diminution of all parts of the body, particularly of the breasts. We have given the reason of it. I have never seen such accidents, at least if the doses have not been too large; but this is no small reason to be very circumspect in employing the new preparations.

*Mode of employing iodine.*

*Tincture of iodine.* ℞ Alcohol at 35°, 1 ounce,
Iodine, 48 grains.

This tincture should not be prepared long before it is used, because it soon deposits the crystals of iodine; there is reason to fear, besides, that the iodine might deprive the alcohol of part of its hydrogen, and thus be converted into ioduretted hydriodic acid.

The tincture of iodine has been used with much success in the cure of goitre; it has also been employed in the treatment of scrophula, but not so often as the two following preparations:

The tincture of iodine is given to adults in the dose of 4 to 6 drops, 3 times a day, in a half glass of sugar and water; it may be augmented progressively up to 20 drops, three times a day; 20 drops contain 1 grain.

*Ioduretted sulphuric ether.*

℞ Sulphuric ether, 1 grain,
Pure iodine, 6 grains.

Thirty drops contain 1 grain of iodine. Patients hardly support more than 10 drops at a time.

*Solution of the hydriodate of potash.*

℞ Hydriodate of potash, 36 grains,
Distilled water, 1 ounce.

This solution is still capable of dissolving iodine, and thus to form an ioduretted hydriodate of potash.

If it is wished to make a solution called Coindet's, it is sufficient to add 10 grains of pure iodine to the solution of hydriodate of potash indicated above.

These two preparations, the mode of administration of which is the same as that of the tincture of iodine, are employed like that in the treament of goitre and scrophula; in the latter case there is usually associated some medical tonic.

*Ointment of hydriodate of potash.*

℞ Hydriodate of potash, 1-2 gros,
Ungt. simplicis, 1 1-2 ounce.

The dose of this ointment, which is used in friction, morning and evening, in goitre and enlarged scrophulous glands, is a demigros for each time. In about eight days it may be carried to 1 gros, and even more, according to the age of the subject and the extent of the tumour.

Sometimes, by this means, a complete resolution is obtained of tumours which the saline solutions have not entirely dissipated.—This ointment has been employed in divers cases of enlargement of the testicles, which have not yielded to the influence of other means; sometimes also, the treatment by friction does not produce a complete cure, and often it is necessary to combine the two means. In general, in the treatment of scrophula, more advantage seems to be derived from the use of saline solutions.

When the method of friction is used in the treatment of goitre, it is well sometimes, to aid the action of iodine by emollient fomentations or leeches. Sometimes, after the first frictions, the goitre, so far from softening, becomes hard, and slightly painful; the application of a few leeches ordinarily causes this local irritation to disappear, and the effects of iodine are then shown in a very marked manner.

This ointment may be made more active by adding 10 or 15 grains of pure iodine, which forms what is called the ointment of ioduretted hydriodate of potash." pp. 90 to 99.

We have administered Iodine to a number of patients afflicted with different maladies; but are not prepared to speak of its powers in the language of eulogy. Our expectations, in fact, have not been realised. The absorption of the mammæ and testes, which its long continued use is said to have occasioned, has not occurred in our practice. Nor has it produced other sinister effects, although administered to a variety of persons in liberal quantities. When not sufficiently diluted, it occasions gastrodynia, without vomiting or stupor; and in large doses would, no doubt, excite acute gastritis; for when externally applied, its irritating effects are extremely violent. We have found an ointment containing only a fiftieth part of its weight of iodine, to smart the skin intolerably, and produce a fiery papular eruption. This is the strength of the ointment of iodine and calomel of Coindet; which, according to our experience, is at least twice as strong as is adapted to practice in this country. We have recommended the tincture of iodine in the scrophulous affections of the negroes of Kentucky, but did not find it successful. Our trials have not, however, been numerous. In a case of chronic bronchitis, attended with dilatation of the cavities of the heart, it afforded some palliation. The patient, a medical gentleman of correct observation, at first took the tincture, and became well acquainted with its taste. At a subsequent time, he used the medicine in substance, in the form of pills, when he recognised its flavour in the mucous which he expectorated—an evidence of its absortion and elimination from the lungs, like the odorous parts of garlic. We have mentioned in the eighth number of the Western Medical and Physical Journal, the beneficial influence of our medicine in a case of scrophulous opthalmia. No return of the malady has since taken place. The same child had a scrophulous tumour of the cheek, which began to suppurate before it entered on the use of the medicine. The suppurative action continued, but the abscess never broke, and seems likely to heal by absorption and granulation. The medicine was used both internally and externally. From the alledged power of Iodine in promoting the absorption of solid parts, we have been induced, in the practice of the Cincinnati Eye Infirmary, to administer it for the removal of the films and nebulæ, which give opacity to the cornea, after violent opthalmias; but its effects, in that way, were not very obvious. We have directed our medicine in one case of irritable testis, of several years standing, unconnected with syphilis or gonorrhœa. For a time it seemed to afford some mitigation; but the ultimate benefit was inconsiderable. To a woman with stricture of the œsophagus, we have administered the Iodine for four months, without any abatement of the malady, or material change in her general health, which was delicate. This patient has a slight enlargement of the thyroid gland; which suffered no diminution during the use of the medicine. In common with some of our brethren, we have, in a spirit of empiricism, directed the tincture of iodine in several other cases, agreeing in little else, than this obstinacy under ordinary plans of treatment; but, on the whole, have not obtained very encouraging results.

The remaining articles of this work are Lupuline, Croton oil, Piperine, Urea, Euphorbia latyris, Lactucarium, Gold and Platina, some of which present matters of interest; but the extent which our review has already attained, admonishes us to come to a termination.

We commend the publication of M. Majendie, for we are not among those who disapprove of the search after new medicines. To look out for new emetics or cathartics, would, it is true, be a work of supererogation; but our therapeutick armories are not limited to the means of promoting evacuation. Many of our most useful articles, are those which produce no sensible effects on the excretory organs, of which Opium, Bark, Arsenic, and Mercury under certain modes of administration, may be mentioned as examples. Even the most beneficial effects of Antimony are, in many cases, almost independent of its evacuating operation. Diseases, on the whole, have a more specific character, than many theorists and system mongers are inclined to admit; and we want a greater number of medicines, possessed of something like a specific power, in reference to particular morbid actions. Such a power, as the Sulphate of Quina, and the Deuto Chloride of Mercury, possess over ague and fever and syphilis. That the catalogue of this class of remedies (if we may be permitted to class them on the principle of their relations to particular maladies) will be greatly augmented, we entertain no doubt; and it is, perhaps, chiefly to chemistry that we must look for the acquisition. No other science is so fertile in both discoveries and creations; and they equally administer to the progress of clinical medicine. It reveals to us things which would forever have been concealed, such as Morphia, Quina and Antimony, while it may fairly be said to create those sulphates and tartrates of these natural productions, which are so precious to us in the practice of medicine. The number and variety of these creations are absolutely without any limits, as the chemists may increase them at pleasure, by the skilful junta-position of known elements. We regard it, therefore, as a justifiable anticipation, that the Materia Medica is destined to be enriched with new articles, until its powers shall far transcend the imperfect resources of the present time.

